# Supplementary material for: Understanding the Online Environment for the Delivery of Food, Alcohol and Tobacco: An Exploratory Analysis of ‘Dark Kitchens’ and Rapid Grocery Delivery Services
Source: Int J Environ Res Public Health. 2022 May 2;19(9):5523. doi: 10.3390/ijerph19095523 (PMC9099441; doi:10.3390/ijerph19095523)
Supplement: Supplementary file 1 [file ijerph-19-05523-s001.zip › ijerph-1690564-supplementary.pdf]

Supplementary Materials

Price comparison between Rapid grocery delivery services and traditional supermarket chains.

The Grocer 33 list is based on the standard grocery basket in the UK. 33 products sold on dark grocer apps and on delivery platforms were compared to the prices of three supermarket chains in the UK with different price points (Sainsbury's, Waitrose & Partners and Morrisons). The websites of supermarket chains were used to access product prices, which correspond to the product prices in-store. All three dark grocer apps identified in this analysis (Getir, Zapp and Gorillas) were included in the price comparison. For grocery businesses on delivery platforms, a selection of three businesses was made (Sainsbury's on Uber Eats, Waitrose & Partners on Deliveroo and Morrisons on Deliveroo). Prices of businesses on food delivery platforms were compared directly to the prices of the same business in-store to create an exact match. This was only possible for supermarket chains on delivery platforms, as convenience stores and off-licenses typically do not have their own website with product prices.

Where possible, the same product brands and packaging sizes were compared. Where this was not specified in The Grocer 33 list, brands and packaging sizes were selected or modified based on what was available on dark grocer apps and food delivery platforms (which is limited compared to in-store for large supermarket chains). The chosen products differed slightly depending on the type of rapid grocery delivery service.

Products used for dark grocer apps: *[Any modification to the original list or further specifications can be found between brackets]*

1. Gala apples *[4-pack]*
2. Baked beans, 400g can *[Heinz brand]*
3. Shampoo *[Head and Shoulders brand, 225ml]*
4. Victoria sponge cake *[also replaced by other whole cake]*
5. Cornflakes *[Kelloggs brand, 450g]*
6. Butter brioche rolls *[St Pierre brand, 300g]*
7. Cheetos cheese puffs *[replaced with Kettle Chips brand]*
8. Chicken *[breast, 380gr]*
9. Chicken goujons *[replaced with other breaded chicken product]*
10. Chopped tomatoes *[Cirio or Napolina brand]*
11. Conchiglie pasta shells *[also replaced with spaghetti; premium brand e.g. Garofolo]*
12. Lemonade *[Sprite brand, 500ml]*
13. Double cream
14. Flora spread *[also replaced with Pure brand]*
15. Fusilli pasta *[premium brand e.g. Garofolo]*
16. Vanilla ice cream *[Ben and Jerry's brand]*
17. Linda McCartney burgers *[also replaced with Meatless Farm brand]*
18. Maltesers *[29g]*
19. Mixed pepper stir-fry *[replaced with 3-pack of peppers]*
20. Mixed vegetables *[replaced with mixed salad/lettuce, 125g]*
21. Mozzarella *[125g, full fat]*

22. Plums *[replaced with grapes]*
23. Pork sausage *[Thorner's brand; 400gr]*
24. Quorn mince *[also replaced with Quorn brand sausages]*
25. Raspberries *[also replaced with blueberries, 125g]*
26. Ruby port *[also replaced with vintage port]*
27. Sponge cloths *[also replaced with sponges]*
28. Corn on the cob *[replaced with canned corn, Green Giant brand]*
29. Tomato puree *[Cirio or Napolina brand]*
30. Tortilla wraps *[Old el Paso brand, 8-pack]*
31. John West tuna chunks in brine *[replaced with Rio Mare brand, 2-pack]*
32. UHT milk semi-skimmed 1L *[replaced with fresh semi-skimmed milk]*
33. Warnurton's wholemeal loaf *[also replaced with Hovis brand]*

Products used for supermarkets on food delivery platforms: *[Any modification to the original list or further specifications can be found between brackets]*

34. Gala apples *[4-pack]*
35. Baked beans, 400g can *[Heinz brand]*
36. Shampoo *[Aussi brand, 500ml]*
37. Victoria sponge cake *[also replaced by whole cheese cake]*
38. Cornflakes *[Kelloggs brand, 450g]*
39. Butter brioche rolls
40. Cheetos cheese puffs *[replaced with Doritos brand cheese flavour]*
41. Chicken *[breast]*
42. Chicken goujons *[also replaced with chicken kievs]*
43. Chopped tomatoes
44. Conchiglie pasta shells *[also replaced with spaghetti]*
45. Lemonade *[sugar-sweetened]*
46. Double cream *[300g]*
47. Flora spread *[also replaced with Pure brand]*
48. Fusilli pasta
49. Vanilla ice cream *[Haagen-Dazs brand]*
50. Linda McCartney burgers *[also replaced with Linda McCartney brand sausages]*
51. Maltesers *[102g]*
52. Mixed pepper stir-fry *[replaced with 2-pack of peppers]*
53. Mixed vegetables *[300g]*
54. Mozzarella *[whole, full fat]*
55. Plums *[400g]*
56. Pork sausage *[400gr]*
57. Quorn mince *[also replace with Quorn brand sausages]*
58. Raspberries
59. Ruby port *[also replaced with Viejo rioja wine]*
60. Sponge cloths *[also replaced surface wipes]*
61. Corn on the cob
62. Tomato puree
63. Tortilla wraps

64. John West tuna chunks in brine *[replaced with own brand canned tuna]*
65. UHT milk semi-skimmed 1L *[replaced with fresh semi-skimmed milk]*
